# A qualitative study of physician perspectives of cost-related communication and patients’ financial burden with managing chronic disease

**DOI:** 10.1186/s12913-015-1189-1

**Published:** 2015-11-25

**Authors:** Minal R. Patel, Khooshbu S. Shah, Meagan L. Shallcross

**Affiliations:** Department of Health Behavior & Health Education, University of Michigan School of Public Health, 1415 Washington Heights, Ann Arbor, 48109-2029 USA; Department of Internal Medicine, University of Michigan Health System, 1500 East Medical Center Drive, Ann Arbor, MI 48109 USA

**Keywords:** Access to care, Financial burden, Communication skills, Doctor-patient relationships, Patient adherence

## Abstract

**Background:**

Patient financial burden with chronic disease poses significant health risks, yet it remains outside the scope of clinical visits. Little is known about how physicians perceive their patients’ health-related financial burden in the context of primary care. The purpose of this study was to describe physician experiences with patients’ financial burden while managing chronic disease and the communication of these issues.

**Methods:**

In November 2013, four focus groups were conducted in an academic medical center. A convenience sample of 29 internal and family medicine resident physicians was used in this study. A semi-structured interview protocol was employed by trained facilitators. Coded transcripts were analyzed for themes regarding physicians’ experiences with identifying, managing, and communicating financial burden with their patients in the context of primary care.

**Results:**

Major themes identified were 1) patient financial burden with chronic care is visible to physicians, 2) patient’s financial burden with chronic care and discussing these issues is important to physicians, 3) ability to identify patients who perceive financial burden is imperfect, 4) communication of financial burden with patients is complex and difficult to navigate, 5) strategies utilized to address concerns are not always generalizable, and 6) physicians have ideas for widespread change to make these conversations easier for them.

**Conclusion:**

Awareness of physician perspectives in identifying and addressing their patients’ disease-related financial burden may better equip researchers and medical educators to develop interventions that aid care teams in better understanding these patient concerns to promote compliance with treatment recommendations.

## Background

Chronic diseases require management with a therapeutic regimen and routine follow-up with clinicians- a common reality for one in two individuals in the U.S [1]. One in five families also perceive financial burden with managing their health care costs [2], with these burdens more common with chronic disease [3].

With exception to select provisions in insurance systems for vulnerable populations, most health insurance plans in the U.S. require individuals and families to pay for various portions of their health care out-of-pocket. Nearly 20 % of U.S. health care costs are paid out-of-pocket by patients. Affordability challenges are among the top four reasons why patients do not follow through with therapeutic recommendations [4]. Financial burden with healthcare costs poses significant risk to patients and society when patients utilize harmful strategies to address these burdens, such as non-adherence, which can adversely impact health status [5, 6]. The apparent consequences of patient’s financial burden with disease management underscores the need for further attention of how disease management can be supported when cost-related barriers may preclude patients from following through with therapeutic recommendations.

Other work suggests that the communication of out-of-pocket costs between clinicians and patients occurs infrequently [7]. Some clinicians may simply be unaware that patients’ out-of-pocket expenses are a problem. Since clinicians make therapeutic recommendations, realizing ways in which care teams can support patients’ disease management in the face of cost-related concerns is a practice priority that has been largely unexplored in a systematic way. We could not locate any studies on how physicians view the potential health-related financial burdens of their patients. Given that so little is known, we conducted a qualitative study that allowed physicians to express their views in their own words. The purpose of this study was to describe physician experiences with patients’ financial burden with managing chronic disease and the communication of these issues. Obtaining information in this unconstrained manner provides a firmer foundation for the subsequent development of interventions to alleviate this pressing public health problem.

## Methods

Qualitative focus groups were used to carry out this research.

### Sample

Participants were a convenience sample of internal medicine and family practice resident physicians at a large academic medical center in the United States that serves half of its state’s population, representing both urban and rural settings across the socioeconomic spectrum. Patients seen at the academic health center are both insured and uninsured and represent a mix of individuals with both private and government sponsored plans. Resident physicians in this setting are compensated with a fixed annual salary. Participants were identified through resident physician rosters of the health system. Inclusion criteria included 1) family practice and internal medicine resident, 2) second or third year in their resident training program, and 3) 18 years of age and older. First year residents were not contacted due to their limited experience providing clinical care. Fifty residents were randomly selected for contact via e-mail to participate in a 90-minute focus group. Thirty physicians agreed to participate and 29 actually attended the focus groups conducted in November 2013. Those who did not agree to participate stated time conflicts as a barrier to participation. Physicians were assigned to focus groups based on their availability to attend within a very limited clinical schedule. Nine participants came to the first focus group, seven to the second, six to the third, and seven to the fourth group. All participants provided written informed consent and were given modest monetary compensation for their participation. All study procedures were approved by the University of Michigan Institutional Review Board for the Social and Behavioral Sciences.

### Focus group design and data collection

Fourteen questions and prompts were developed as part of the moderator’s guide. Table 1 shows examples of questions from the discussion guide.

**Table 1:** Examples of questions from the focus group discussion guide

First I want to ask how frequently patients with chronic disease bring up issues around affordability with you. Does this happen often? Sometimes? Never? Remember, these conversations can be as simple as a patient expressing concern about the costs of a medication, which isn’t covered by insurance. What is the culture around having these conversations in your practice or the delivery system in which you work?
When a patient talks about their out-of-pocket expenses related to their chronic illness, what topics do they bring up? How confident are you in answering these questions?
How important do you think it is to have a conversation about out-of-pocket expenses with your patients? [Probe: If it helps, you could think of it relative to other tasks you have to perform.]. If you don’t think it’s very important, who should patients have these conversations with?
What barriers get in the way of you speaking with your chronically ill patients about out of pocket costs?
From your observations, how are patients’ cost concerns impacting their ability to manage their health?

Each focus group was led by two facilitators, the primary author, and a research assistant. All discussions were audio-recorded. Participants completed informed consent and a survey of demographic and clinical practice information. Consistency of procedures was ensured across the groups through the use of the standardized interview protocol and systematic training of facilitators. All facilitators established ground rules for discussion, encouraged spontaneous discussion, clarified concepts and questions, and actively solicited participation from less-vocal participants [8]. Data saturation was reached after four focus groups.

### Analysis

To check for completeness of the transcription, a review of the focus group transcripts and audio-recordings was conducted. Preliminary codes (first level codes) were generated based on a review of each transcript to identify statements reflecting recurring distinct themes regarding experiences with financial burden of patients with chronic diseases. Second level codes were then created. Codes were refined as needed and transcripts were checked to ensure that coded data reflected the refined codes. Reliability was assessed by checking transcripts for obvious mistakes and spot-checking data with codes during the coding process to ensure that there was no drift in the code definitions [9]. NVivo 9 software was used to organize codes.

All transcripts were coded by two independent coders who met to resolve coding differences to enhance reliability. Interrater reliability for the categories was assessed using Cohen’s Kappa and was found to be appropriate for exploratory work (k = 0.79). Coded questions were used to generate themes regarding physician views of patient financial burden with managing chronic disease and strategies for intervention.

## Results

Table 2 describes sample characteristics (*n* = 29). Six distinct themes emerged from the focus group discussions (Table 3).

**Table 2:** Demographic and clinical practice characteristics of focus group participants (*n* = 29)

Variable	Percent (*n* = 29)
Age (Mean (SD))	28.55 (2.01)
Male	62 %
Race/ethnicity	
White	55 %
Black	4 %
Asian	31 %
Other	10 %
Year in post-graduate training	
PGY 2	76 %
PGY 3	24 %
Medical Specialty	
Internal medicine	93 %
Family medicine	7 %
Time spent per week providing direct patient care	
≤30 h	4 %
31–39 h	10 %
40 or more hours	86 %
Percentage of patients seen with chronic conditions	
50–79 %	34 %
≥80 %	66 %

**Table 3:** Major domains identified on physician perceptions of patients’ financial burden with managing chronic disease

Domain	Description	Example
Patient financial burden with chronic care is visible to physicians	Physicians see issues of financial burden often in their chronic disease patients, especially with COPD and diabetes. They notice that these burdens go beyond the out-of-pocket costs with medicines. Physicians feel worried about behaviors patients engage in to manage their financial burden.	“One of my patients will stretch out their Lantus and they will make it last like 45 days or 2 months. Then they wonder why they’re like poorly controlled and have to keep raising their dose.” (male second year internal medicine resident)
Patient’s financial burden with chronic care and discussing these issues is important to physicians	Physicians view patients’ financial burden as important because it impacts compliance, and believe these issues will continue and magnify with the Affordable Care Act.	“I think if you assess that it’s gonna affect whether or not they actually take the med, then it’s probably one of the most important things because you can tell them all of these recommendations but they’re not gonna do em.” (male third year internal medicine resident)
Ability to identify patients who perceive financial burden is imperfect	Physicians have a hard time identifying patients who may be at risk for forgoing care due to cost, they have never been trained to address these issues, and they make assumptions based on limited information.	“I had a couple of admissions, COPD exacerbations…they had not been taking their maintenance inhalers or something like that and it turns out that it was ‘cause they couldn’t afford it.” (male second year internal medicine resident)
Communication of financial burden with patients is complex and difficult to navigate	Physicians experience discomfort in having conversations with patients about affordability and financial burden. Social distance, perception of no solution, lack of training and cognitive burden preclude them from initiating these conversations.	“It’s a disaster, there’s not formalized training about how to navigate that conversation or how to set a follow-up conversation to happen in the future with their primary care physician or whatever. That is totally lost to me.” (male second year internal medicine resident)
Strategies utilized to address concerns are not always generalizable	Physicians utilize $4 and $10 generic lists, recommend low cost pharmacies in the area, and recommend splitting higher dose pills to reduce financial burden. Physicians acknowledge that these strategies may not be generalizable.	“For the most part, I feel like it’s easy to find a lot of alternatives on the list. And that is the main place that I go at least when someone brings up cost issues is the $4 or the $10 lists ‘cause usually that’s hopefully not an issue, and you can usually find an acceptable alternative.” (female second year internal medicine resident)
Physicians have ideas for widespread change to make these conversations easier for them	Physicians recommend utilizing ancillary care staff, incorporating questions into screening forms, and leveraging the electronic health record to better identify and address patients’ financial burden.	“And I think those screening tools are super helpful for us physicians to flag the social issues that we maybe don’t see and don’t talk about.” (female second year family medicine resident)

### Domain 1: patient financial burden with chronic care is visible to physicians

Physicians are aware of the financial burden of managing chronic disease among their patients. Nearly three quarters of the sampled physicians mentioned that more than half of the patients they see have affordability concerns. There was consensus across groups that these issues are seen often in both sub-specialty and primary care clinics, especially among individuals managing diabetes, COPD, or multiple chronic conditions. There was also consensus that medications and diabetes management supplies are among the most common sources of financial burden for patients. However, beyond medications and supplies, physicians also mentioned that expenses associated with traveling to clinic visits, such as gas and parking, were common concerns. Physicians often hear from their patients that recommended ancillary services such as nutrition counseling, physical therapy, or mental health services become costly due to limited insurance coverage for such services.

Participants expressed their concern regarding some of the measures they see their patients taking to address their financial burden with managing their condition. Reduction in the frequency of taking meds and sharing medicines among family members were particularly concerning as noted by participants:

“The concern is they’re supposed to be under medications that’s twice per day and they’re only taking it once. It’s like well, we’re just going in circles because there’s a reason for most meds that it’s twice a day because of its half-life” (male third year internal medicine resident)

### Domain 2: patient’s financial burden with chronic care and discussing these issues is important to physicians

Most of the sampled participants expressed that patient’s financial burden is important to them, and believe it is their responsibility to address:

“I think it is our responsibility because we’re the people who can determine a) if you can get it and b) do you need this medication or can you switch to a different kind that would be cheaper” (female second year internal medicine resident)

Many physicians expressed that they see patient’s out-of-pocket costs as a barrier to compliance and therefore an important factor in patients’ ability to follow through with treatment recommendations that they provide. However, some physicians felt that they have higher clinical priorities with patients than cost, and struggled with how to fit affordability into their practice framework as noted by a male participant:

“It’s important to emphasize value to me….yeah, that’s sort of how I think about it and that leaves me a little bit puzzled about how to think about affordability in this particular way.” (male third year internal medicine resident)

Physicians noted that they believe that issues of affordability for patients will remain and will be magnified with the Affordable Care Act, given the influx of the newly insured and the wide range of insurance options that continue to be available through both the public and private sector. The Affordable Care Act does not eliminate out-of-pocket payments from individuals, but rather provides more programs and subsidies to lower out-of-pocket expenses, and provides more comprehensive coverage for essential medicines and services.

### Domain 3: ability to identify patients who perceive financial burden is imperfect

Participants’ ability to identify patients who perceive financial burden or may be at risk appears imperfect. The majority of participants mentioned that affordability is not the first thing that comes to mind for them when they recommend a treatment to patients because they have not been trained to keep that in mind for the patient or consider it a priority. They emphasized that no more than a single day in their education has been devoted to learning about payment structures, identifying financial burden as a barrier to noncompliance, and keeping care affordable for patients. Some patients are reluctant to bring up the issue, thus also contributing to difficulties in identifying the issue.

However, there was consensus across groups that affordability challenges come up very often with their patients, and it occurs when the issue of noncompliance comes to light, which is often during follow-up visits, readmission, or after an urgent care episode; long after treatment recommendations are made. One participant noted that sometimes it comes up at the expense of almost alienating the patient:

“a grad student who had type 1 diabetes and our attending went to go see this patient and essentially berated her for not talking her insulin for the last three days. …it was because she ran out of insulin and she didn’t have enough money to buy the insulin. …we always assume that when it’s not being filled or they’re not taking it, it’s because they just want to be noncompliant. …But a lot of times, it might be from cost, we just don’t know it.” (female second year internal medicine resident)

Physicians identified patterns that they see among patients where the issue of cost and affordability is brought up. They noticed that the newly insured, individuals with Medicare, and those with private insurance bring up the issue of cost frequently. Some physicians also mentioned they notice that even very nominal co-pays with Medicaid can be taxing on patients who have very limited income. However, some physicians’ reflection of their experiences revealed overreliance on limited information or observation of the patient in identifying and believing whether financial burden was truly a barrier to carrying out treatment recommendations. One participant reflected on one of their patients:

“it’s like you have a brand name purse, you have an iPhone, you smoke two packs a day and you’re telling me you can’t afford $8 a month for medication” (male second year internal medicine resident)

### Domain 4: communication of financial burden with patients is complex and difficult to navigate

Most participants mentioned that they never or rarely communicate financial burden with their patients, and nearly three-quarters report that when these issues do surface, it is their patients who are initiating the conversation. Participants felt that it was easiest to have conversations about affordability with patients when they know the patient is uninsured. There was consensus among physician participants that the communication of financial burden between them and their patients is a patient prompted conversation under most circumstances, and the reasons why they do not initiate the conversation are multifaceted. Participants mentioned that discussing affordability with patients felt like a taboo subject because there is no formalized training about how to initiate that conversation. Participants also felt that the discussion of financial burden often ends up being a very prolonged conversation, and lack of time during the visit precludes them from bringing it up. They also feel cognitively burdened by their current responsibilities as a physician.

Many participants expressed low self-efficacy and discomfort with these conversations. One source of these feelings is lack of a solution to offer the patient. One female participant noted:

“I feel bad that I can’t help them and I find that to be very uncomfortable. I don’t have an answer and I don’t know how to get you an answer and you’re here asking me for help that I can’t provide. I find that to be very uncomfortable.” (female second year internal medicine resident)

Another source of discomfort stemmed from the socioeconomic distance between physicians and their patients, especially in the context of patient affordability challenges. Some physicians felt conflicted on how to effectively engage the patient in a productive conversation in this context while trying to maintain rapport as articulated by one participant:

“…I don’t ever want to come off as condescending in my interactions with the patient- but it is a little condescending to be prescribing expensive therapies as someone who can afford it to someone who can’t. And I think that it’s difficult to tell someone you need these medications. You need this treatment. Oh, you can’t afford it? Oh, I can afford it. And I think that creates an uncomfortable kind of gap in the patient/physician relationship.” (female third year internal medicine resident)

### Domain 5: strategies utilized to address concerns are not always generalizable

Physicians who have engaged with patients about affordability and financial burden mentioned several strategies they have utilized to address these concerns. The most common strategy mentioned was the use of the $4 and $10 lists of generic medicines available through most pharmacies. Some physicians also mentioned that they will prescribe a medicine at a higher dose if it is the same price, and have the patient cut the pills. They also encourage their patients to utilize mail-order pharmacies, as well as certain pharmacies in town that offer the best prices.

Participants also expressed that these strategies are sometimes not helpful in certain practice situations. They mentioned that patients at shelters and clinics in low-resource settings have affordability challenges even with low cost generic lists. They also acknowledged the limitations of pill cutting with certain types of medications such as those that are inhaled or come in gel caps. Some participants felt comfortable eliminating medications from patients’ regimens that the physicians believed provided little additional value to their health, while many others expressed discomfort in making decisions about non-essential medicines when the regimen is composed of prescriptions from a variety of specialties.

### Domain 6: physicians have ideas for widespread change to make these conversations easier for them

Participants were asked about strategies that would be helpful to them to make cost-related discussions easier to implement within the framework of their clinical care load. Many participants felt that ancillary care providers such as complex care managers, social workers, or medical assistants were better skilled and most helpful in addressing patients’ financial burden. However, some participants acknowledged that ancillary staff are not always available in some practice settings. Some participants noted that they would like to see more capability with the electronic health record to flag affordability concerns, and show more transparent reporting of the total out-of-pocket cost to patients for prescribed treatments and services. Others suggested that pre-clinic forms would be useful in flagging patient affordability concerns so that they can refer to them to start a conversation.

## Discussion

To our knowledge, this is the first study that has used focus groups to gather an in-depth understanding of physician perspectives of their patients’ financial burden with managing chronic disease. In the six domains that emerged, we found that although physicians are aware and place importance on patients’ financial burden as a risk factor in following through with treatment recommendations, they face difficulties identifying those who are at risk and effectively communicating affordability issues with patients.

Because physicians have difficulty identifying patients at risk for cost-related non-adherence and they face discomfort in discussing affordability with patients, these treatment concerns may be overlooked. Merely observing the patient to make inferences about affordability may miss individuals who are at significant risk for not following through with treatment recommendations as a result of affordability challenges. Often, assets that presume affordability or affluence do not necessarily correlate with income, disposable income, or access to financial resources to pay for treatment recommendations at a given point in time. Income and access to financial resources can be fluid, or the patient may not control or be the decision-maker of household finances. These scenarios have implications for clinician-patient rapport and underscore the need to have better diagnostic tools that aid care teams in objectively uncovering patients’ affordability context to facilitate access and compliance with treatment recommendations. Given that affordability challenges are often uncovered during or after adverse and expensive medical episodes such as emergency department visits and hospitalizations, early detection is a practice priority.

Physicians’ reluctance to discuss affordability and financial burden with their patients may stem from the fact that up until recently, financial burden as a side-effect of treatment has been largely ignored in the practice of medicine and physician training [10, 11]. The social distance often evident between clinicians and patients make these conversations difficult to initiate and navigate [12]. Balancing ethical and moral principles inherent in the culture of medicine in providing care of best clinical value to the patient is also difficult with the practical realities of true access for the patient. Compounding these difficulties are the lack of evidence-based approaches that have been widely integrated in health care for identifying and systematically addressing patients’ affordability concerns and financial burden to promote compliance.

There are limitations to this study that should be noted. This was an exploratory study with a small number of participants intending to generate qualitative descriptions of physicians’ experiences with patient financial burden. However, the size of the sample was consistent with other work employing qualitative approaches with physician participants [13, 14]. The study sample comprised resident physicians practicing in an academic health system. Findings may not be generalizable to physicians in all practice settings and geographies. Given that resident physicians are still training, they may be less likely than established physicians to attend to non-medical issues. However, this sample comprised resident physicians who are at the later stages of their training. Since they typically spend more time with patients than attending physicians and rotate through a variety of specialties and practice settings through which chronic care is provided, their perspective may not be that different from established physicians. This study was limited to understanding physician perspectives and may leave out important beliefs, values and behavioral practices of patients that are important to understand in the context of financial burden and care-seeking priorities.

Despite these limitations, this research provides important information on gaps in patient-centered care and identifies areas for behavioral interventions and research to make conversations about affordability more common in clinical practice in order to promote compliance, especially in the context of long-term chronic disease management. In health care systems where high and low cost treatment options is negligible, the issue of financial burden may still be relevant for preventive health care practices outside of care-seeking. Recent reforms related to reducing out-of-pocket spending for patients in the United States under the Affordable Care Act are anticipated to have variable impact among individuals and families due to nuances in the law and anticipated care-seeking behaviors of the newly insured [15]. Discussing sensitive clinical topics between clinicians and patients have advanced in the areas of sexual health, drug use, and pain management. They may provide useful intervention models for thinking about how financial burden can be integrated into clinical care and patient and medical education to promote compliance. Although the cost of therapies is not within the control of individual practitioners, solutions to address patients’ financial burden in providing patient-centered care have been described in the literature based on practice experience [16, 17]. One of these solutions is providing generic equivalents that may be a beneficial practice for all patients irrespective of ability to pay. Further investigation on the utility, generalizability, and wide-spread use of such solutions is a research priority to aid physicians and clinical practice teams in providing patient-centered care.

## Conclusion

Although physicians recognize that patients’ financial burden with out-of-pocket costs can have implications for adherence to therapeutic recommendations, they face difficulties navigating these conversations and having accessible strategies to help their patients. Awareness of physician perspectives with patients’ disease-related financial burden may better equip researchers and medical educators to develop interventions that aid care teams in better understanding these patient concerns to promote compliance with treatment recommendations.
